# “He Doesn’t Listen to My Words at All, So I Don’t Tell Him Anything”—A Qualitative Investigation on Exposure to Second Hand Smoke among Pregnant Women, Their Husbands and Family Members from Rural Bangladesh and Urban India

**DOI:** 10.3390/ijerph13111098

**Published:** 2016-11-08

**Authors:** Cath Jackson, Rumana Huque, Veena Satyanarayana, Shammi Nasreen, Manpreet Kaur, Deepa Barua, Prashanta Nath Bhowmik, Mithila Guha, Mukesh Dherani, Atif Rahman, Kamran Siddiqi, Prabha S. Chandra

**Affiliations:** 1 Department of Health Sciences, University of York, Heslington, York YO10 5DD, UK; cath@validresearch.co.uk (C.J.); kamran.siddiqi@york.ac.uk (K.S.); 2 ARK Foundation, House 130, Road 21, New DOHS, Mohakhali, Dhaka 1206, Bangladesh; rumanah14@yahoo.com (R.H.); shammibadrul@yahoo.com (S.N.); deepa31oct@gmail.com (D.B.); parthonsu@gmail.com (P.N.B.); mithilaguha@gmail.com (M.G.); 3 National Institute of Mental Health and Neurosciences, Bangalore 560029, India; veena.a.s@gmail.com (V.S.); mksnimhans@gmail.com (M.K.); 4 Institute of Psychology, Health and Society, University of Liverpool, Liverpool L69 3GL, UK; M.K.Dherani@liverpool.ac.uk (M.D.); Atif.Rahman@liverpool.ac.uk (A.R.)

**Keywords:** smoking, tobacco, second hand smoke, middle income, pregnancy, women, fetus, smoke free homes, low and middle income countries

## Abstract

Second hand smoke (SHS) exposure during pregnancy is associated with poor pregnancy and fetal outcomes. To design interventions to reduce exposure, an in depth understanding of social and cultural factors of smoking behavior at home is important, especially in South Asia where SHS exposure is high. This study aimed to explore pregnant women’s, their husbands’ and other family members’ knowledge, attitudes and practices regarding home SHS exposure. Semi-structured interviews were conducted with 33 participants in Comilla, Bangladesh and 31 in Bangalore, India (36 pregnant women, 18 husbands, and 10 family members). Data were analyzed using the Framework approach. Husbands smoked in various living areas inside the home, often in the presence of their pregnant wives. Most had never tried to stop smoking at home. Knowledge of the risks was generally poor. Most women had repeatedly asked their husband to smoke outside with little success and only few family members had reprimanded the husbands. Husbands who had stopped did so because of requests from children and their mother. Potential strategies to decrease SHS exposure at home were educating the husband about risks and supporting the pregnant women in negotiation. Interventions must also enlist family support to enhance the woman’s self-efficacy.

## 1. Introduction

### 1.1. Why Is Second Hand Smoke a Problem for Women?

Smoking and smokeless tobacco are leading threats to global health among both men and women [1]. The risks to women in several parts of the world, particularly Europe, Western Pacific and Southeast Asian regions, are greater from second hand smoke (SHS) exposure with estimates that more than one third of women above 15 years are regularly exposed to SHS in their homes [2,3]. Data from the Global Adult Tobacco Survey India, 2009 [4] and Bangladesh Demographic Health Survey 2011 [5] suggest that nearly 45%–50% of women are exposed to SHS at home.

Health risks including lung cancers, coronary heart disease, stroke, chronic obstructive pulmonary disease, oral and pancreatic cancer have been reported as a consequence of exposure to tobacco smoke [6,7,8,9]. More than 80% of deaths among women in low and middle income countries (LMIC) are due to cardiovascular diseases, cancer and chronic obstructive pulmonary disease, which account for 50% of the total disease burden [2,10,11].

Pregnant woman are particularly vulnerable to SHS. Studies from China and India have shown poor pregnancy outcomes such as preterm deliveries, and poor infant outcomes specifically low birth weight, small for gestational age infants, still births and sudden infant death syndrome [12,13,14,15,16,17,18]. Exposure to household SHS in early infancy also increases severe infectious morbidity requiring hospital admission [12].

### 1.2. What Are the Particular Challenges for Women?

Women have a higher rate of exposure to SHS than men, especially in South and Southeast Asia. In China, of all women who are exposed to SHS, 90% of them have exposure at home and 60% have spouses who smoke regularly at home [19,20]. Gender inequity and gendered power interactions have been found to mediate smoking behaviors within homes in these countries. SHS exposure is more among women from rural areas and those with low education. Studies done in Cambodia, China and India highlight women’s inability to negotiate smoke free homes [21,22]. This is true even when women are pregnant [22]. Factors such as lack of awareness, attitudes towards smoking, and cultural factors such as respect for elders that prevent negotiation, also lead to SHS exposure at home [14,15,16,17,23]. Studies in India, China and Cambodia have also shown that the knowledge of perceived health risks of SHS is limited among women exposed to SHS [24]. These studies highlight how despite holding negative attitudes towards smoking, Chinese women choose not to articulate their opinions as it would disturb the equilibrium at home. They might even rationalize men’s smoking behavior as it indirectly contributes to the family income by increasing the husband’s work and social networks [25].

### 1.3. What Do We Know about Interventions to Reduce SHS Exposure in Pregnant Women?

Several strategies have been tried to decrease exposure to SHS among pregnant women. These include establishing and encouraging women to enforce smoke free home rules, enhancing their self-efficacy, increasing awareness, providing knowledge, changing attitudes and developing assertiveness skills [13,23]. A significant increase in knowledge, a change in attitude and an increase in assertiveness skills among pregnant women when exposed to SHS at home have been shown following these interventions [22,23]. The Trans-Theoretical Model and its stages of change may offer potential for informing interventions to prevent exposure to SHS [23].

Community based interventions including advice and community educational programs have been some methods that have been tried and found to be successful [26]. In Indonesia, wives of smoking husbands stated that their husbands would comply with a community initiative to decrease home smoking [24]. Developing interventions that target men may thus relieve the pressure off a woman to confront male authority figures at home [27]. Empathetic and sensitive interventions targeting both men and women are more likely to be positively received in traditional Asian countries where social harmony is important [28].

### 1.4. Why Do We Need to Do This Study—What Are the Gaps in the Literature?

Pregnancy is considered a “teachable moment” when families are willing to make changes for the well-being of the fetus. Despite some research on reducing exposure to SHS in pregnancy in LMIC, some gaps exist. There are a lack of data on what men and women know about the effects of SHS on pregnancy and the fetus; the barriers and facilitators to smoking outside the home among men; and the attitudes of other family members in joint and extended families to smoking when there is a pregnant woman at home. Family support for the pregnant women in ensuring a smoke free home; cultural and social factors that prevent pregnant women from enforcing smoke free home rules and men and women’s ideas about what kind of interventions would work are also poorly understood.

Qualitative approaches allow an in-depth understanding of the richness and the complexity of the social and cultural factors that influence smoking behavior at home when women are pregnant. This is important for designing appropriate interventions during this period [24]. The aim of this qualitative interview study was to explore pregnant women’s, husbands’ and other family members’ knowledge, attitudes and practices that promote or inhibit SHS exposure in the home. A secondary aim was to seek feedback on draft materials for a multi-component behavior change intervention targeted at pregnant women to enable them to negotiate a smoke free home with their husbands. This multi-component behavior change intervention has now been developed; its feasibility and preliminary efficacy in preventing home exposure to SHS will be evaluated in a pilot trial with a sample of pregnant women in India and Bangladesh. The intervention was informed by findings from this qualitative study, a modified Delphi study and a systematic review (papers in preparation). The four components of the intervention are: a letter from the unborn fetus to the father, cotinine feedback, a picture booklet and mobile phone based voice messages.

## 2. Methods

### 2.1. Ethics

The study was approved by the Institutional Ethics Committees of the University of Liverpool, the National Institute of Mental Health and Neurosciences (NIMHANS/DO/99th IEC/2015) and the Bangladesh Medical Research Council.

### 2.2. Study Design

This was a qualitative study. It was the first “intervention development” stage of the Medical Research Council framework for developing and evaluating complex interventions [29]. This stage is used to inform the components of the intervention and the mode of delivery [29].

### 2.3. Setting and Participants

The research was undertaken in two sites: Comilla, Bangladesh and Bangalore, India. Comilla is a typical peri-urban district about 100 km southeast of the capital, Dhaka, with a population of 5.4 million. The literacy rate in the district is about 55%, and the economy is mainly based on agriculture and cottage industries [30]. There are approximately 200 community clinics in the district, which are staffed by Community Health Care Providers to deliver an essential service package for women, children and the poor. Four community clinics with at least 100 pregnant women registered during the study period were selected for the study.

The Bangalore study site was an antenatal clinic located in the South Zone of Urban Bangalore city catering to 32 urban slums in the Bruhat, Bangalore, Mahanagara and Palike municipal areas. The total population of these slums is around 149,743 with a ratio of 927 females per 1000 males. Most of the women (91%) are homemakers while the remaining are engaged in unorganized labor (home maids). The majority of the men (75%) are employed in the unorganized sector and both unskilled and skilled labor, such as carpenters, plumbers, auto rickshaw drivers are daily wage labor.

We set out to interview approximately 30–40 participants in both sites (total 60–80 participants). We purposively sought to recruit a mix of pregnant women whose husbands smoke in the home (*n* = 8–10 per site), pregnant women whose husbands no longer smoke in the home (*n* = 8–10 per site), husbands who smoke in the home (*n* = 5–7 per site), husbands who no longer smoke in the home (*n* = 5–7 per site) and family members of husbands who smoke in the home (*n* = 4–6 per site). We were confident that this sample size would enable us to look for potential differences and similarities in the views of pregnant women, husbands and family members; as well as draw out meaningful comparisons across the two sites. A final inclusion criterion was that the participant needed to agree for the interview to be digitally recorded.

### 2.4. Recruitment

In Comilla, the Community Health Care Providers first contacted the pregnant woman and requested her to visit the community clinic with her husband and a family member. The researcher then met in a private space with the woman to check eligibility (self-reported current/previous SHS exposure, consent to the interview being recorded), go through an information sheet about the study, secure written consent and conduct the interview. For those pregnant women or husbands/family members who could not attend the community clinic, a researcher visited their house. The same approach was followed for interviewing husbands and family members, ensuring that conversations occurred away from others to maintain privacy. Participants were given an allowance of Taka 50 (1 U.S. dollar) to thank them for their time. A leaflet containing information on the adverse effects of SHS was provided after completion of the interview.

In Bangalore pregnant women who attended the antenatal clinic for their regular check-up were assessed for eligibility by the researcher in a private space while they were waiting to see the doctor. Those who were eligible were given an information sheet about the study and written consent was sought before conducting the interview. Some women requested 24 h to consent while others consented on the same day. Husbands were either approached as they waited for their wives outside the antenatal clinic or via their pregnant wives. Family members who accompanied the eligible pregnant women to the antenatal clinic were also approached. The researcher took care to ensure all conversations occurred away from others to maintain privacy. All participants received an information sheet and provided written informed consent before commencing the interview. They were given a food packet to thank them for their time.

### 2.5. Data Collection

Face-to-face interviews were conducted by the local research teams in the community clinic or the participant’s home (Comilla), and at the antenatal clinic (Bangalore). The interviews occurred from December 2015 to February 2016. Participants were interviewed individually. The researchers were novice interviewers. They were trained by Cath Jackson to conduct the interviews using the topic guide, focusing on asking open questions as well as using prompts and probes. Detailed feedback was provided on each interview transcript to continually improve interviewing skills. All interviews were digitally recorded. The interviews lasted between 15 and 90 min.

Topic guides for the interviews (pregnant women, husbands, family members) were developed to ensure consistency across the two sites, although the format was flexible to allow participants to generate naturalistic data on what they viewed as important. The topic guides were piloted in Comilla and questions were subsequently re-ordered to improve the flow of the interview and re-worded to aid comprehension. An overview of the interview topics is presented in Table 1. The focus of the interviews was the husband’s smoking, however when participants spoke of other family members’ smoking in the home this was also explored.

### 2.6. Data Analysis

Interviews were translated and transcribed verbatim and the data subjected to thematic analysis using the Framework approach [31] which is designed to address applied policy and program-related questions. All transcripts were first checked for accuracy by the researcher who conducted the interview to enhance rigor and to ensure that the local context in which the data were collected was retained. The following stages of Framework analysis were undertaken by a team of researchers in the UK, Bangladesh and India. A data analysis protocol was developed to ensure consistency across the research team. NVivo 10 (QSR International, Warrington, Cheshire, UK) and Excel 2010 software packages (Microsoft Corporation, Redmond, WA, USA) facilitated data management.

*Familiarisation*: All the research team read the interview transcripts to record emerging ideas and recurrent themes that were relevant to the aims of the study.

*Constructing a thematic framework*: A thematic framework for the pregnant women interview data was developed using the interview topic guide and eight interview transcripts (four from each site). The framework was applied to a further four transcripts (two from each site) and refined as necessary. Two further frameworks were developed in the same way for the husband and family member interview data.

*Indexing and Charting*: The three thematic frameworks were then systematically applied to the interview data. On the rare occasion when the data did not easily fit into the framework, the “other” category within in each theme was used to ensure that these data were captured. Charts were produced in NVivo for each theme and summaries of responses from participants and verbatim quotes were entered. A sub sample of the completed charts for each participant group in each site was reviewed by C.J. to check the detail and sufficiency of the summaries and quotes.

*Mapping and Interpretation*: The completed charts were exported from NVivo into Excel. These were then reviewed and interrogated to compare and contrast views, seek patterns, connections and explanations within the data. At this point similarities and differences across the two research sites and participant groups (pregnant women, husbands, family members) were explored. Descriptive Findings documents were written up for each participant group focusing on the knowledge, attitudes and practices that promote or inhibit SHS exposure in the home; and views on the proposed interventions for achieving a smoke free home. The research team in each site then reviewed these documents to: (1) check that the interpretation of the local data reflected the intended meaning spoken during the interviews; and (2) where necessary provide local context.

## 3. Findings

### 3.1. Participants

We completed 33 interviews in Comilla and 31 interviews in Bangalore. As intended, in both sites we achieved a mix of pregnant women whose husbands (no longer) smoke in the home, husbands who (no longer) smoke in the home and family members of husbands who smoke in the home (see Table 2). However we did not meet our target sample size for husbands who no longer smoke in the home (both sites) or for women whose husbands who no longer smoke in the home (Bangalore). The reasons offered for declining to take part were that husbands mostly smoked outside the home, a lack of time, feeling unwell, fearful of repercussions of participating in the study (pregnant women), not smoking in the home or not interested (husbands) and not knowing about the husband’s smoking behavior (family members).

### 3.2. Views of Pregnant Women, Husbands and Family Members on SHS Exposure in the Home

The findings from the interviews with pregnant women, husbands and family members are presented below. These themes and sub-themes emerged from the thematic frameworks. The focus of the study was the smoking behavior of husbands of pregnant women however where family members’ smoking behavior is mentioned, these accounts are presented. For some themes and sub-themes, all three participant groups offered their views so all perspectives are presented. For others, only one or two of the participant groups spoke of the issue and so only those accounts are included. Where there are differences in views by research site, participant group or by whether the husband (no) longer smokes in the home, these are highlighted. Illustrative quotes are presented throughout.

### 3.3. Smoking Behaviors

All participants brought up the issue of smoking behaviors of the husbands and their family members (predominantly men: brothers/brothers-in-law, fathers and fathers-in-law). Some of the family members interviewed were unable to comment on the husband’s smoking (see theme *How Do Other Family Members React to the Husbands Smoking in the Home?*).

How can I tell that? He stays outside most of the time. He goes out early in the morning and returns home around 12 to 1AM. How will I know?

(CF06: Family Member, Comilla)

The majority of the men smoked cigarettes, less smoked bidis or both cigarettes and bidis. (Bidis are a thin, Indian cigarette filled with tobacco flake, wrapped in a dried leaf and are a traditional method of tobacco use throughout South Asia). Accounts, from the women and the husbands themselves, suggested that most husbands smoked over 10 cigarettes/bidis a day, whilst about half of them smoked more than 20 a day. This was irrespective of whether they smoked in the home or not.

He smokes a lot. He can smoke a whole packet of cigarettes in a day.

(BWS02: Woman whose husband smokes in the home, Bangalore)

Those husbands who smoked in the home smoked in several rooms, specifically the living room, kitchen, bathroom, bedroom as well as in the passage and on the terrace. Often this was in the presence of others, particularly their pregnant wives. Many smoked when children, members of the family and friends were around.

Does he smoke in the presence of others at home?

(Interviewer)

No… only in front of me will he smoke.

(BWS07: Woman whose husband smokes in the home, Bangalore)

Usually I smoke in front of my wife and a child who reads in class 3. When they stay in front of me while I am smoking, I maintain a distance from them within the room.

(CHS06: Husband who smokes at home, Comilla)

The women and husbands also mentioned several places away from the home where the husbands smoke, namely in an outdoor passage, on the road, at the bus stop, at the shops or tea stalls, at work and at social gatherings. For some men this was in addition to smoking at home, for others an alternative location.

My husband’s a farmer so he spends most of his time during the day at the fields and that’s when he smokes usually.

(CWS03: Woman whose husband smokes in the home, Comilla)

#### Husbands’ Previous Attempts to Stop Smoking in the Home

Most of the women whose husbands currently smoked in the home stated that he had never tried to stop doing this. Only a small minority described how their husband had quit (sometimes just temporarily) smoking inside, prompted either by a significant life event, for example getting married or by her “scolding”.

Has your husband ever tried to stop smoking inside the house?

(Interviewer)

He did, for instance, when we got married, then initially when I’d ask him to not smoke in front of me, he’d go outside. Now, gradually, he smokes more inside the house than previous times.

(CWS01: Woman whose husband smokes in the home, Comilla)

Similarly, most of the husbands who continued to smoke at home reported that they had never tried to stop. Amongst them, one said that whilst he had never tried, he did not think that it would be difficult to do. Only one husband described his experience of trying to stop smoking in the home, thwarted by his addiction.

I decided several times to stop smoking at home…But, due to addiction I start smoking unmindfully again…I was unable to control myself.

(CHS 04: Husband who smokes in the home, Comilla)

Several husbands discussed their attempts to quit smoking, rather than smoking in the home. One said that he had stopped smoking for a year when he was in Saudi Arabia because he was in the desert and there were no shops selling cigarettes. Another stated that he had stopped smoking for six months because he had heard that smoking was bad for his health. Being addicted, and in the company of friends and colleagues who smoke, were seen as the key barriers to quitting smoking. Family members also tended to speak of husbands of pregnant women trying to quit smoking rather than stop smoking in the home, describing short periods of time where they had quit.

### 3.4. Knowledge of the Risks of Smoking and of SHS 

Most of the participants appeared to understand that the husband’s or a family member’s smoking was harmful to the smoker’s own health. Several women and family members described symptoms that they had observed in the husbands, most commonly mentioning a cough and chest pain, as well as weight loss and headaches. Similarly, many husbands spoke of the damage to their own health of smoking, complaining of a cough or headache, as well as chest pain and stomach ache. Longer term risks of smoking were identified as kidney problems, heart problems, breathing problems, lung disease, tuberculosis and cancer. A small minority of the women stated that they knew that their husbands’ smoking was bad for his health, but they were unsure how.

*I think it’s harming him. He’s lost a lot of weight, after smoking he doesn’t feel hungry so he suffers from gastric problems… He complains of headaches and it’s cold he complains of a cough.*

(CWS03: Woman whose husband smokes in the home, Comilla)

Knowledge of the risks of SHS to the pregnant woman was less evident, with almost unanimous unfamiliarity with the concept of a “safe distance”. Several of the women and family members acknowledged that the husband’s smoking in the home was affecting the health of the pregnant woman, describing most frequently how the woman had a cough, but also suffered from headaches, feeling nauseous, dizziness and breathing difficulties. However, only one of the husbands who currently smoked in the home said that he knew that his smoking affected his wife’s health although he did not know how.

No one told me anything about that [SHS]. Even I don’t know about that. Since very beginning am smoking. I always smoke. Till today they never said anything about side effects of cigarette.

(BHS04: Husband who smokes in the home, Bangalore)

Knowledge of the risks of smoking to the unborn baby was also limited. None of the husbands could describe the risks to their unborn baby. A few women and family members commented that the husband’s smoking was dangerous for the unborn baby. Although very few could explain how the baby might be affected, typically mentioning the baby’s breathing.

I heard that smoking creates problem in breathing in children. The smoke of the cigarette when enters into the body of the pregnant woman affects the child in the womb.

(CF04: Family member, Comilla)

#### Sources of Information and Advice about Smoking and SHS

The television was the key source of information about the risks of smoking and SHS in the home, although a small number of husbands stated that they did not believe these messages.

I feel these things will not happen in real life.

(BHS02: Husband who smokes in the home, Bangalore)

Other sources were newspapers, books, school, cigarette packets, advertisements on a notice board at the hospital and other members of the community. Only a small minority stated that their doctors informed them of ill effects of SHS from their husbands’ smoking.

I got to know about it from doctors. Many doctors told me that smoking is harmful. They suggest to stay away from it.

(CWS12: Woman whose husband smokes in the home, Comilla)

### 3.5. Reasons That Husbands Smoke in the Home

The reasons identified for the husbands smoking in the home reflected both the advantages of smoking inside, as well as the disadvantages of smoking outside. Many women and husbands spoke of the husband liking to relax by smoking in the comfort of their own home, often in front of the television (sometimes sat with the children), with a cup of tea or in bed just before going to sleep as his pregnant wife lay next to him. For some husbands this time to relax was seen as important because they felt stressed or sad.

We can’t sit outside and relax, it’s comfortable inside.

(BHS04: Husband who smokes in the home, Bangalore)

I smoke in front of my wife. Usually she takes a rest whilst I smoke at night.

(CHS03: Husband who smokes in the home, Comilla

I don’t like to smoke at home, but after coming home from work, I will be tired and too many tensions so I smoke.

(BHS03: Husband who smokes in the home, Bangalore)

A small minority of the husbands explained that they smoked in the home because, prior to taking part in this interview, they had not been aware of the risks of SHS to their family. Whilst several women and family members commented that the husband’s addiction to smoking led him to smoke in the home.

The key disadvantage of smoking outside was perceived as the fear of being seen by other people, notably the neighbors and elderly relatives; but most significantly the husband’s parents who were often considered not to know of their son’s smoking.

Actually he is scared of his parents, especially his mother. That’s why he doesn’t smoke infront of his mother. He smokes inside the room and keeps the door closed.

(CWS10: Woman whose husband smokes in the home, Comilla)

Before smoking I check out whether my parents are around me or not. Actually my parents do not know that I smoke.

(CHS05: Husband who smokes in the home, Comilla)

Other reasons for not smoking outside, offered by individual women or husbands, were the cold temperatures in winter, a fear of snakes and insects in the evening, concerns about safety outside of the compound, having nowhere to go to smoke outside and the fines for smoking in public places or for being outside after 10:00p.m.

They [Government] will put fine, so I will smoke in the home only.

(BHS03: Husband who smokes in the home, Bangalore)

### 3.6. Reasons That Husbands No Longer Smoke in the Home

The most common reason for husbands no longer smoking in the home was the influence of other people, particularly children, asking him to smoke outside. As an example, one husband stated that he had stopped smoking in the home because his daughter kept breaking his cigarettes.

Since my daughter asked me to stop smoking, I do not smoke inside the home anymore. She does not like me smoking cigarettes. She crushed cigarettes several times.

(CHNS02: Husband who no longer smokes in the home, Comilla)

Some of the women whose husbands had quit smoking in the home attributed this to the husband responding to her feelings of nausea from the smell of smoke now that she was pregnant.

I explained to him and he listened. Yes, I forbid him. I asked him why he smokes at home in front of the baby. We cannot tolerate the smell. After that day, he started smoking outside the home and never smoked inside the home again and I never saw him smoking in front of me either.

(CWNS06: Woman whose husband no longer smokes in the home, Comilla)

Others believed it was the persistent requests from their husband’s relatives, often his mother, which had worked. Indeed, having more than one person in the family telling him to smoke outside was perceived by many women to be helpful.

I had returned from prayers and I saw him smoke in our house. I got upset and angry and asked him to stop and then went to speak to my mother-in-law about the same. Then she came and spoke to my husband and told him: “Don’t smoke in front of her can’t you see she’s uncomfortable? She can’t tolerate the smell of the smoke. If you want to smoke, smoke outside but not inside anymore. She told you that she has difficulty breathing when you smoke. What if an accident happens and something happens to her? Who will be responsible then?” Ever since then, he’s never smoked indoors.

(CWNS01: Woman whose husband no longer smokes in the home, Comilla)

Alternative reasons for no longer smoking in the home, offered by two husbands, referenced an understanding of the risks associated with SHS as being a motivating factor. One of these men, who no longer smoked at home, stated:

Since my wife has a baby in her womb now, I decided to stop smoking in front of her at home… A new baby is developing in its mother’s womb. That’s why I chose not to smoke at home in front of her. If she feels like coughing, she will get ache in her stomach.

(CHNS01: Husband who no longer smokes in the home, Comilla)

### 3.7. How Do Pregnant Women React to Their Husbands Smoking in the Home?

Many women talked about disliking the smell from their husband’s smoking in the home. Other complaints were having difficulty in breathing and feeling nauseous.

The smell is awful … and when I am with him I inhale the smoke through my nose and mouth.

(CWS09: Woman whose husband smokes in the home, Comilla)

I felt sick, was unable to eat properly… I feel very bad. I can’t breathe properly when I inhale the smoke of cigarettes.

(CWS12: Woman whose husband smokes in the home, Comilla)

The majority of women whose husbands continued to smoke in the home described how they had repeatedly told their husbands not to smoke in front of them or their children, requesting him to smoke outside, in another part of the home or indeed to quit smoking completely. Some had tried to explain to the husband the risks of SHS, one had sought support from her mother-in-law and one woman broke up her husband’s cigarettes whenever she found a packet of cigarettes. Many spoke of feeling fed up with asking because their husbands ignored them completely, told the woman to leave the room herself or to leave the family home, or simply smoked inside the next time. This had led to some women choosing to no longer challenge their husband’s smoking in the home.

He doesn’t listen to my words at all, so I don’t tell him anything, Even though I tell him not to smoke, he will never listen.

(BWS01: Woman whose husband smokes in the home, Bangalore)

Whenever I say not to smoke in the home he will listen to me for that moment but later he smokes so I am fed up with telling him again and again, now I am feeling very bad to tell him.

(BWS03: Woman whose husband smokes in the home, Bangalore)

Several women spoke of being frustrated that they were the only one asking the husband to smoke outside, for some this was because family members were not present when the husband smokes or because family members chose not to discuss it with the husband.

If I say not to smoke at home then he will scold and I am the only person to tell and my in laws also stay away from me, if they are with me then he would have not smoked at home so I am finding it difficult to tell him.

(BWS07: Woman whose husband smokes in the home, Bangalore)

Similar accounts were provided by most of the husbands who continued to smoke in the home, as well as by family members who had observed conversations between pregnant women and their husbands. Many husbands did not believe that it was hard for their wives to request them to smoke outside. They said that they did not get angry, with a few explaining that it is the wife’s duty to advise him on the risks of his smoking. Most admitted that they did not listen or only sometimes listened to their wives’ frequent requests.

It is not difficult for her at all. No, I did not get angry at her. When she asks me to quit smoking, I feel that she is telling this for the betterment. She is discouraging me towards smoking in order to ensure a good health.

(CHS03: Husband who smokes in the home, Comilla)

I did not give much importance to my wife’s words.

(CHNS02: Husband who no longer smokes in the home, Comilla)

Sometimes I move in the different room to smoke.

(CHS06: Husband who smokes in the home, Comilla)

A minority of husbands said that their wives did not react to their smoking in the home. Similarly, a small minority of women stated that they would not ask their husband to smoke outside. For a few, this was because they were scared that their husband would be angry. One woman said that she was frightened to ask her husband to smoke outside as she was fearful that he would send her back to her parents without her son.

So, being a short-tempered person, he gets angry very fast, if anyone tells him anything. And once he gets angry, he’ll begin to throw things around and break them. So that’s why I don’t tell him much.

(CWS09: Woman whose husband smokes in the home, Comilla)

Another preferred that he smoked in the home as she was concerned that he would drink alcohol with his friends if he went outside.

If he smokes at home, he will be under control to smoke or drink, but if he goes out with friends he will not be in control, he smokes a lot and drinks very “heavy”. So I will not send him outside to smoke.

(BWS02: Woman whose husband smokes in the home, Bangalore)

### 3.8. How Do Other Family Members React to the Husbands Smoking in the Home?

Several different reactions of family members were described. Many women and husbands who continue to smoke in the home said that other family members would not know about this, because the husband did not smoke in front of them.

Does he smoke in the presence of others in the family?

(Interviewer)

He won’t smoke when relatives visit. They do not know.

(BWS03: Woman whose husband smokes in the home, Bangalore)

Others suggested that family members were aware of the husband’s smoking behavior, but chose to not say anything. Several possible reasons for this behavior were offered: they had not seen it for themselves, believed that the husband needed to smoke for his addiction or stress, were frightened to confront the husband, did not believe in the risks of SHS or because the father or father-in-law smoked in the home. In some cases, it was the woman’s belief that her husband’s family pretended to not know about his smoking in the home.

*She [husband’s mother] never saw him doing it but has heard about it. She says “I can’t talk to him about this since I have never seen him doing that in front of me”.*

(CWS11: Woman whose husband smokes in the home, Comilla)

Yes they [husband’s parents] know, but they don’t say anything about it… He doesn’t smoke in their presence he only smokes in my presence. They don’t say anything to him, but they say “let him smoke he will be having tensions”.

(BWS01: Woman whose husband smokes in the home, Bangalore)

A small number of women and husbands spoke of family members having reprimanded the husband for smoking, as well as for smoking in the home. Most the husbands appeared to have ignored this, although for one, this had prompted him to stop, on the request of his father.

Whenever he [brother] calls me, he gives advice to quit smoking and always pray to Allah. He does not know that I smoke inside the home.

(CHS05: Husband who smokes in the home, Comilla)

Everyone in our family is asking him to quit smoking. If he doesn’t listen to them, what they can do about it?

(CF04: Family member, Comilla)

Finally, a few husbands mentioned that their children repeatedly asked them to quit smoking in the home.

### 3.9. What Would Help Husbands to Quit Smoking in the Home?

When asked, what would help the husbands to quit smoking in the home two ideas strongly emerged: the influence of other people’s requests and the husband understanding the risks of his smoking to his unborn baby. However, there were mixed views about the potential impact of both strategies.

The common view was that the husband most respected the views of his mother or both parents. A few identified the husband’s siblings or mother-in-law as the key influence. A small minority of the women, and of the husbands themselves, stated that he respected no one. For one husband this was because he was the guardian of the village.

There is no one [who I respect]. I am the elder person now and also the guardian of this village. Most of the old people of this village die. Besides I am the Judge of the village court.

(CHS06: Husband who smokes in the home, Comilla)

Sometimes this respect was relevant to the husband’s smoking behavior, in that the husband hid his smoking from that person (see theme *How Do Other Family Members React to the Husbands’ Smoking in the Home?)*, or this person was viewed as someone who could successfully influence where he smoked.

Would you stop, if your parents asked you to stop smoking inside the home?

(Interviewer)

Yes, I would stop smoking. If my parents were informed about this, I would stop smoking because I cannot hurt my parents.

(CHS03: Husband who smokes in the home, Comilla)

A different view, mainly evident amongst the women and family members, was that despite this respect, the husband would ignore requests to smoke outside. Indeed, some mothers of husbands described how their son did not listen to them about smoking, or would promise to smoke outside and then break this promise later.

It is not difficult [to ask the husband to smoke outside], he is my son. He does not listen to me when I forbid him.

(CWS04: Woman whose husband smokes in the home, Comilla)

Consistent with the accounts of the husbands who no longer smoked in the home (see theme *Reasons that Husbands No Longer Smoke in the Home)*, requests by children were seen as the most likely reason that husbands would smoke outside. This was suggested by women, husbands and family members.

He listens to his children most. His children are always forbidding him and hence, he does not smoke in front of them. In absence of his children he smokes inside the home.

(CF04: Family member of a husband who smokes in the home, Comilla)

Most the women were hopeful that if their husbands were informed of the risks of SHS to her and the unborn baby, then he would stop smoking in the home because he would not want to hurt his child. The consensus was that this education would need to focus particularly on the risks to the fetus, as to date the husbands had not typically responded to their wives’ complaints about smoking near her (see theme *How Do Pregnant Women React to Their Husbands Smoking in the Home?)*. Most of the husbands similarly commented that they would be motivated to stop smoking at home if they knew that there were risks of SHS to their unborn child, and their children.

I think if someone convinces him properly and tells him to stop smoking for his wife and unborn child, he may follow the advice little bit for the baby.

(CWS11: Woman whose husband smokes in the home, Comilla)

The source of the information was seen by some as important. The majority view being that a professional, for example a university employee or health professional, would be viewed as more credible than the wife and hence have more impact on the husband’s behavior.

What would be the best way for your husband to receive this [SHS information]?

(Interviewer)

If you go then it will be very good. More than me, perhaps he could give more importance to what you have to say.

(CWS01: Woman whose husband smokes in the home, Comilla)

A small minority of the husbands were clear that they would not act on the advice of a doctor, because they knew that their doctor smoked himself. Finally, two other strategies that were offered by individual husbands as potentially helpful were managing the husband’s stress and the husband applying some “self-control”.

## 4. Discussion

To our knowledge, this is the first study to explore the experiences and beliefs related to SHS exposure among pregnant women from LMIC which included pregnant women, their husbands and family members. Bloch et al. [32] discuss how interventions for tobacco exposure for pregnant women in LMIC should be preceded by research that dissects the unique social and cultural issues related to tobacco smoking, specifically in the context of gender relationships and power differentials. They further mention that studies need to involve not just the individual woman but the household, community, and the health care setting. Our study followed this recommended method for intervention development. Other strengths were the use of one-to-one interviews which facilitated in-depth discussion of knowledge, attitudes and practices that promote or inhibit SHS exposure in the home; and the use of software for data coding and analysis, providing an auditable pathway from the primary data to the final findings. Triangulation was achieved by involving pregnant women, husbands and family members from households where husbands had stopped smoking in the home, as well as those where husbands continued to smoke inside. This enabled us to explore differences and similarities in these participant groups; as well as look for meaningful comparisons across the two sites (Comilla and Bangalore). The main limitation was that we that struggled to recruit participants, particularly husbands, who lived in homes where there was no longer SHS exposure and those accounts are under-represented.

Our key findings were: (1) a sense of helplessness among most women from both Comilla and Bangalore in negotiating smoke free homes alongside the feeling that other family members did not support her in this; and (2) a lack of knowledge among women, husbands and family members about the effect of SHS on the pregnant woman or her unborn baby, with almost absent information and advice from health professionals. Together these promoted SHS exposure in the home and inhibited the creation of smoke free homes. The factors that seemed to help husbands to stop smoking in the home were concerns about the health of the unborn baby, the health of other children in the household and being pressurized from the entire family to smoke outside.

The sense of helplessness amongst the women was striking and this parallels the findings of studies from South and Southeast Asia which have similarly observed women’s inability to negotiate smoke free homes [21,22,25]. These authors attribute this to gender inequity and gendered power interactions. Greaves and Hemsing [33] also emphasize how gendered roles, relationship patterns, and caring responsibilities influence women’s experience of the family, household, and domestic spheres and may prevent discussion of SHS exposure in pregnancy or otherwise. They caution against the common focus of SHS policies and programs on the health of children, or, in the context of pregnancy, on the exposure of the fetus to SHS, emphasizing that such approaches may actually reproduce traditional gender roles and de-legitimize women’s health. It is interesting that most women in our study mentioned that the husband would stop smoking for the children, or for the health of the unborn baby rather than for his wife’s health. Careful consideration is needed to develop smoke free interventions during pregnancy that can address the woman’s own health as well as fetal health without undermining her sense of agency. Our intervention prioritizes the health of the unborn baby and other children; with the health of the pregnant woman as an important secondary focus (we present feedback on the women’s cotinine levels). A pilot trial will now assess the acceptability and usefulness of this approach. In terms of our related finding that women feel unsupported in their endeavor to negotiate with their husband, our intervention booklet that is given to the women invites the entire family to act together. Involving the wider family to present a “united front” in negotiating a smoke free home echoes the recommendation by Nichter and colleagues [24] that any intervention should include the whole family, and could usefully extend further to engaging the wider community.

Poor knowledge about SHS and the risks to the health of the fetus and children are consistent with the findings of a recent systematic review [34] that explores the barriers, motivators and enablers to smoke free homes. The review had a broader focus, i.e., it was not focused on pregnancy and drew on studies based predominantly in high income Western countries [34], which suggests that the need for interventions to include educational components are not unique to our study population. For our participants, the perception from the pregnant women was that educating the husbands would trigger an emotional response because they care for their unborn baby, thus leading to them smoke outside. We have therefore decided to present the husband with a letter from the unborn fetus describing the impact of his/her future father’s smoking on his/her development. Advice from a health professional, particularly a doctor, has previously been identified as a motivating factor to smoke away from the family [34] and accounts from participants in this study suggest that there is scope for developing the role of this “credible source” in Bangladesh and India. Indeed, our intervention will be delivered in antenatal clinics by health professionals. The pilot trial of the intervention is now underway.

## 5. Conclusions

The study findings offer valuable insight into the key components and modes of delivery [29] to enable the development of socially and culturally appropriate interventions to prevent or reduce exposure to SHS among pregnant women in LMIC. Interventions that provide methods (such as booklets) by which the woman can negotiate smoke free homes with her husband and family, coupled with simple ways of indicating SHS exposure to the husband (such as a cotinine levels report), may be helpful.

## Figures and Tables

**Table 1:** Overview of Interview Topics.

Topic	Question
Smoking in the home	Who smokes, what do they smoke, when and where? Who do they smoke in front of?
Last time the husband smoked in the home	When and where did he last smoke? How did the woman/husband/family member feel? How did the wife/family member react? Did the wife/family member ask him to go outside? Why/why not? What happened? What are the barriers to asking the husband to smoke outside? What would help the woman/family member to ask the husband to smoke outside?
What would help/has helped the husband to smoke outside	Has the husband previously tried to smoke outside? What are the barriers to the husband smoking outside? What would help the husband to smoke outside? Who can influence the husband’s smoking behavior?
Smoking knowledge	What is the impact of smoking on the health of the husband/pregnant woman/unborn baby? Where did they learn this?
Smoke Free Home intervention	Are they interested in having a smoke free home? What do they think of the four proposed components of a smoke free home intervention? What are the barriers/facilitators to taking part in the intervention?

**Table 2:** Overview of Participants.

Participants	All	Husband Smokes in the Home	Husband No Longer Smokes in the Home
**Pregnant Women**			
Comilla	18	10	8
Bangalore	18	14	4
**Husbands**			
Comilla	9	7	2
Bangalore	9	8	1
**Family Members**			
Comilla	6	6	N/A
Bangalore	4	4	N/A
